# Efficacy and Safety of Recombinant Human Adenovirus Type 5 (H101) in Persistent, Recurrent, or Metastatic Gynecologic Malignancies: A Retrospective Study

**DOI:** 10.3389/fonc.2022.877155

**Published:** 2022-04-28

**Authors:** Jing Zhang, Qiying Zhang, Zi Liu, Juan Wang, Fan Shi, Jin Su, Tao Wang, Fei Wang

**Affiliations:** Department of Radiation Oncology, The First Affiliated Hospital of Xi’an Jiaotong University, Xi’an, China

**Keywords:** gynecologic malignancies, oncolytic virus, recombinant human adenovirus type 5, H101, efficacy, safety

## Abstract

**Background:**

To assess the efficacy and safety of recombinant human adenovirus type 5 (H101) in patients with persistent, recurrent, or metastatic gynecologic malignancies.

**Methods:**

The study retrospectively enrolled patients with persistent/recurrent/metastatic gynecologic malignancies who received H101-containing treatment at The First Affiliated Hospital of Xi’an Jiaotong University from September 1, 2019 to September 30, 2021. H101 was injected intratumorally into target lesions and dosage was calculated based on tumor diameter once a day for five consecutive days. The primary endpoint was local control (LC) rate. Secondary endpoints included objective response rate (ORR), duration of response (DOR) and progression-free survival (PFS). Safety was the exploratory endpoint. Depending on prior treatment, patients received H101 either as monotherapy or as a combination therapy.

**Results:**

Totally, 29 patients were enrolled in the study. Median follow-up was 6.3 months (range: 3.2-27.9) from data analysis cut-off on December 31, 2021. The LC rate at 3 months was 44.8%, while ORR was 72.4%. Median DOR and PFS rates were not determined. The DOR rate, PFS rate at 6 and 12 months were 88.1%, 74.6% and 70.5%, 62.2%, respectively. Responses were observed in all four cancer types. Most treatment-related adverse events (90.5%) were grade 1 or 2, with the most common being fever (70%). Clinically significant adverse events were uncommon (7.9% in grade 3 and 1.6% in grade 4). No treatment-related deaths occurred.

**Conclusion:**

Our study showed that H101 (either monotherapy or combination therapy) has promising efficacy and favorable safety in patients with persistent, recurrent, metastatic gynecologic malignancies.

## Introduction

Patients with persistent, recurrent, and metastatic gynecologic malignancies have poor outcomes and low cure probability (1–4). Treatment options for this population are often limited to systemic therapy (5–7) and their responses rates are relatively lower (2, 3, 8, 9). Recent studies have demonstrated that targeted agents (9–11) and immune checkpoint inhibitors (12–14) exert antitumor activity in multiple tumor types, including gynecologic malignancies. However, persistent, recurrent, or metastatic gynecological malignancies are often present in a single or isolated lesion. These patients often received multiple chemotherapy or high-dose radiation treatments, which reduced the quality of their health and result in less benefit from systemic therapy. Therefore, local therapy is more likely to be an effective strategy, in which precision radiotherapy is an important option for local therapy (15–17). Unfortunately, for the patients who had received radiotherapy before, it is difficult to achieve satisfactory prescription dose again.

Oncolytic virus (OVs) therapy is one of the most promising new anti-tumor therapies and is being increasingly used in the clinic with favorable results (18, 19). There are various modes of administration for OVs therapy, including intratumoral injection, intravenous delivery, and pleural, peritoneal, and intravesical administration (20–22), of which local intratumoral injection is the most standard delivery route. These viruses can selectively replicate in tumor cells, inducing oncolytic responses in their targets and stimulating immune response that attracts more immune cells to kill the remaining tumor cells (23, 24).

Recombinant human adenovirus type 5 (H101) is the first commercialized OVs in the world. The gene encoding the 55 kDa E1B protein responsible for p53 binding and inactivation in H101 has been deleted to confer p53-selective replication of OVs, thereby inducing p53 accumulation, resulting in direct and selective cytotoxicity in tumor cells during replication (25). In 2005, H101 was approved by the National Medical Products Administration (NMPA) of China for treating nasopharyngeal carcinoma. Moreover, the virus has also exhibited anti-cancer properties in head and neck carcinoma and gastric cancer (26, 27), but its effect on gynecologic malignancies remains unclear. Here, this study aimed to assess the efficacy and safety of H101 mono- or combination therapy in patients with persistent, recurrent, metastatic gynecologic malignancies, which provide a new strategy for the treatment of persistent, recurrent, or metastatic gynecologic malignancies.

## Materials and Methods

### Patients

The study retrospectively reviewed patients with persistent, recurrent, metastatic gynecological malignancies who completed at least one cycle of H101-based treatment of The First Affiliated Hospital of Xi’an Jiaotong University from September 1, 2019 to September 30, 2021 and it was approved by the Ethics Committee of The First Affiliated Hospital of Xi’an Jiaotong University. Informed consent was obtained from each participant.

The inclusion criteria were: 1) Age ≥ 18 years old; 2) Recurrence and metastasis of gynecological malignancies confirmed by pathological diagnosis; 3) Had at least one assessable and injectable (under direct vision or palpation) lesion according to Response Evaluation Criteria in Solid Tumors version 1.1 (RECIST 1.1).

The exclusion criteria were: 1) Received previous immune checkpoint inhibitor therapy within 1 month; 2) Hepatitis B virus, human immunodeficiency virus infection or tuberculosis in the active phase or conditions requiring corticosteroids/other immunosuppressive medications; 3) Undergoing other clinical studies and has an unpredictable outcome effect on this study; 4) Unable to tolerate and cooperate with treatment; 5) Refusal to receive H101 treatment.

### Data Collection and Processing

Patient characteristics were obtained from the electronic medical records system of our institution. Variables included age, Eastern Cooperative Oncology Group (ECOG) Scale of Performance Status score, disease diagnosis, pathological type, disease status at enrollment, number of cycles using H101, treatment modalities in combination with H101, cycle number of prior systemic chemotherapies, number of treatment lines, and site of target lesions. Patients were removed if they failed to complete at least one cycle of H101 treatment, could not be followed up on time, or were lost to follow-up.

As one treatment cycle, H101 was injected daily into target lesions (found *via* direct vision or palpation) for five consecutive days and three weeks for a cycle. The maximum tumor diameter determined the injection dose of H101: 5.0 × 10^11^ virus particles (VP) for tumor diameter ≤ 5 cm, 1 × 10^12^ VP for tumor diameter between 5 to 10 cm, and 1.5 × 10^12^ VP for tumor diameter > 10 cm. If lesion residua were present after the first cycle, a new cycle was performed (doses were adjusted based on changes in target lesions) until the lesion disappeared, the disease progressed, or uncontrollable adverse reactions occurred. Patients received either H101 alone or in combination with radiation and other medications, depending on their specific conditions.

### Clinical Assessment

Changes in the target lesions were evaluated using magnetic resonance imaging (MRI) or computed tomography (CT), and were reviewed by the same imaging specialist. Local control (LC) was defined as disappearance of the target lesion, based on gynecological examination or imaging. Tumor response was assessed using RECIST 1.1. Objective response rate (ORR) was defined as the percentage of all patients with complete response (CR) and partial response (PR). Time to response (TTR) was defined as latency from start of H101 treatment to first objective tumor shrinkage based on gynecological examination. Duration of response (DOR) was defined as latency from TTR to date of documented progression (confirmed by gynecological examination/imaging), death, or termination of follow-up. Progression-free survival (PFS) was defined as duration from first H101 dose to first documented tumor progression or death from any cause.

### Safety Assessment

Treatment-related adverse events (AEs) were recorded from initiation to 30 days after the last treatment and the AEs were monitored according to the National Cancer Institute’s Common Terminology Criteria for Adverse Events (CTCAE, Version 5.0).

### Follow-Up

Follow-up occurred two weeks after H101 injection. Then, once a month for three times, and every three months in two years. All patients underwent gynecological examination, MRI or CT imaging, and laboratory examination through out-patient follow-up. Clinical information, response to the treatment, and AEs were recorded at each follow-up.

### Statistical Analysis

All data analyses were performed with SPSS version 22.0 (SPSS Inc. IBM Corp). Demographics, baseline characteristics, and safety data were summarized using descriptive statistics. Categorical data were presented as frequencies and percentages, while continuous data were presented as medians and ranges unless otherwise noted. Time-to-event variables (DOR and PFS) were calculated using the Kaplan-Meier method. The LC rate, ORR, and DCR with 95% CI were calculated using the Clopper-Pearson method. Statistical significance was set at *P*<0.05.

## Results

### Baseline Characteristics

**Figure 1:**
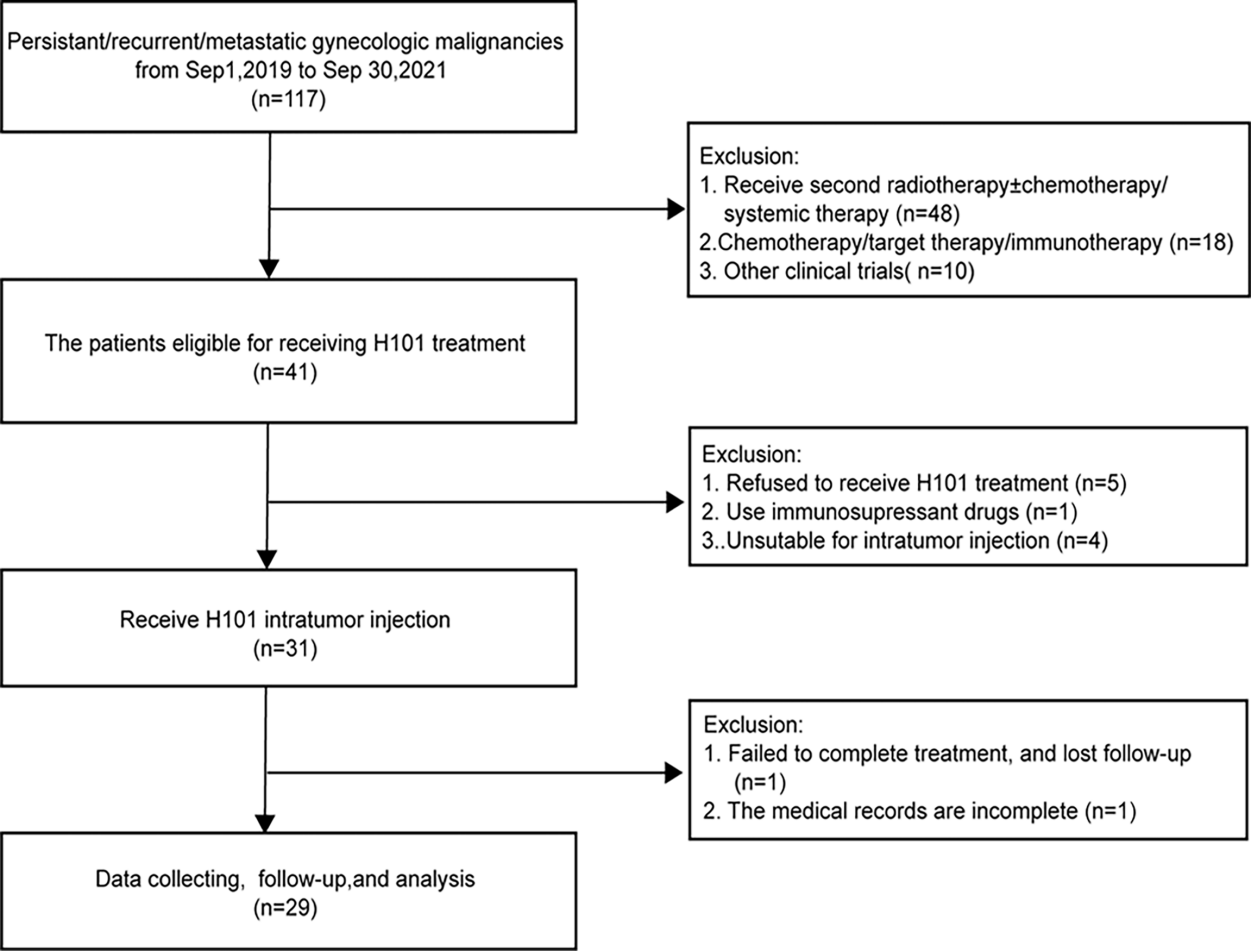
Flow chart of patients entered into the study.

A total of 29 eligible patients were enrolled in this study (**Figure 1**). Patients had a median follow-up time of 6.3 months (range: 3.2-27.9) from the initial H101 treatment to the analysis cut-off date (December 31, 2021). Their median age was 51 years (range: 20-75). The four types of gynecological malignancies included cervical cancer (22 cases), vaginal cancer (2 cases), vulvar cancer (3 cases) and ovarian cancer (2 cases). 16 patients who received H101 treatment due to persistent lesions after radical concurrent chemoradiotherapy were diagnosed with cervical cancer. The rest were recurrent or metastatic gynecological tumors, including cervical cancer (6 cases), vaginal cancer (2 cases), vulvar cancer (3 cases), and ovarian cancer (2 cases).

All patients were treated with two or more treatment lines. 16 patients (59%) received only one cycle of H101 treatment across five consecutive days. Seven patients received H101 monotherapy, while the remaining 22 received H101 combined with radiation and/or other medications (including chemotherapeutic agents, molecular targeted therapeutic drugs, or immune checkpoint inhibitors). All target lesions were intratumorally injected with H101 under direct vision or palpation; 14 (48%) lesions were cervical, 10 (34%) were vaginal, 3 (10%) were vulval, and 2 (7%) were at other sites. Baseline characteristics were shown in **Table 1**.

**Table 1:** Baseline characteristics.

Patient Characteristics	N (%)
Total enrollment	29 (100)
Age (years)	51 (20-75)
ECOG performance-status score^a^	
0	0 (0)
1	22 (76)
2	4 (14)
3	3 (10)
Disease diagnosis	
Cervical cancer	22 (76)
Vaginal cancer	2 (7)
Vulvar cancer	3 (10)
Ovarian cancer	2 (7)
Pathological type	
Squamous cell carcinoma	18 (62)
Adenocarcinoma	9 (31)
malignant melanoma	2 (7)
Morbid state at enrollment	
Persistence	16 (55)
Recurrence or Metastasis	13 (45)
Cycle number of using H101	
1	16 (55)
≥2	13 (45)
Combination therapy	
Only H101	7 (24)
Combination radiation^b^	7 (24)
Combination other medications^c^	8 (28)
Combination radiation plus other medications	7 (24)
Number of therapy lines	
2	17 (59)
≥3	12 (41)
Injection site	
Cervix	14 (48)
Vagina	10 (34)
Vulva	3 (10)
Others^d^	2 (7)
Cycle number of previous systemic chemotherapies	
<4	11 (38)
4-6	10 (34)
>6	8 (28)

Data are No. (%) unless otherwise indicated.

a Eastern Cooperative Oncology Group (ECOG) performance-status scores on a scale from 0 to 5, with higher scores indicating greater disability.

b Including external beam radiation therapy, brachytherapy, or coalition of these two.

c Including chemotherapeutic agents, molecularly targeted agents, immune checkpoint inhibitors, or combinations of the above.

d One is in the umbilical region, and another is in the abdominal wall.

### Efficacy

**Figure 2:**
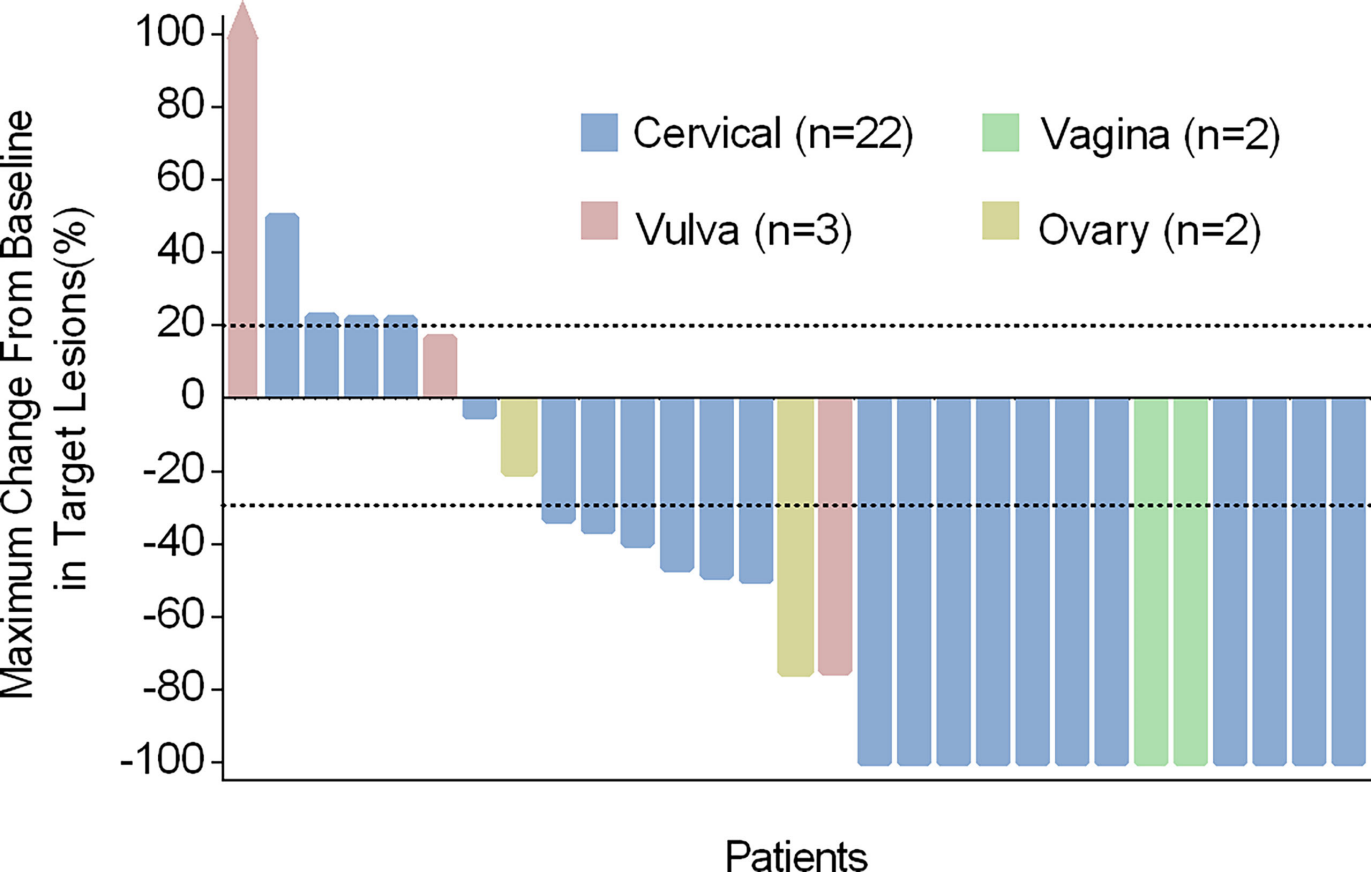
Characteristics of treatment response in different cancer types. Maximum change from baseline in the sum of target lesion diameters. Tumor reduction (bars) corresponds to maximum change from baseline in target lesions and not to the best overall response.

Target lesions disappeared fully in 13 of 29 patients, and the LC rate at 3 months was 44.8% (95% CI: 25.6%-64.1%) and among which the LC rate of H101 single-agent was 17.2% (5/29). 21 patients (13 with CR and 8 with PR) obtained the best overall response and the ORR was 72.4% (95% CI: 55.1%-89.7%) (**Table 2**). Meanwhile, each of the 29 patients underwent one or more radiological and gynecological evaluations, and the optimal percentage change in lesion size from baseline was shown in **Figure 2**. Responses to treatment were observed in all four tumor types enrolled.

**Table 2:** Treatment Responses.

Response	No. (%)
Best overall response	
CR	13 (44.8)
PR	8 (27.6)
SD	3 (10.3)
PD	5 (17.2)
ORR, No. (%; 95CI)^#^	21 (72.4; 55.1-89.7.)
DCR, No. (%; 95CI)^#^	24 (82.8; 68.1-97.4)
Median TTR (days)	4(3-15)
Median DOR (months)	NR (0.6-18.1)

Data are No. (%) unless otherwise indicated.

^#^based on the Clopper and Pearson method.

CR, complete response; PR, partial response; SD, stable disease; PD, disease progression; ORR, objective responses rate; DCR, disease control rate; NR, not reached; TTR, time to response; DOR, duration of response.

**Figure 3:**
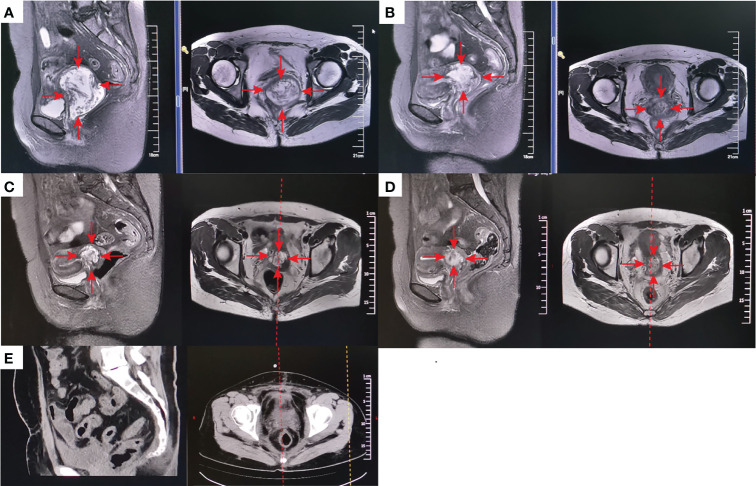
A patient with stage IB3 at the initial diagnosis of mucinous adenocarcinoma of the cervix developed into a huge residual mass of 57^*^76^*^66mm (MRI) **(A)** because of the treatment was terminated by grade 4 myelosuppression due to one cycle of neoadjuvant chemotherapy, which also made the patient refuse concurrent chemotherapy. After external-beam radiotherapy (EBRT) 30Gy plus 1 cycle local injection of H101 (1.0×10^12^ virus particles daily for 5 days), the mass was significantly reduced to 46^*^51^*^48mm (MRI) **(B)**. The mass was further reduced by 32^*^46^*^45mm (MRI) **(C)** after 50Gy of EBRT. After the following brachytherapy (6Gy for 1 time of intraluminal brachytherapy and 7Gy for 3 times of interstitial brachytherapy), the residual lesion was 29^*^39^*^30mm (MRI) after 1 month of radiation **(D)**. Since refusal of chemotherapy, she accepted 1 cycle treatment of H101 (5.0×10^11^ virus particles daily for 5 days) again. Gynecological examination indicated that the lesion had further shrunk, while there were still some suspicious and hard masses. Therefore, surgical resection of the residual lesion was suggested. Postoperative pathology indicated that no cancer cells were found in the surgically excised lesions. One-month postoperative Computed tomography (CT) scan showed no signs of tumor **(E)**. The red arrow stands for the tumor mass.

**Figure 4:**
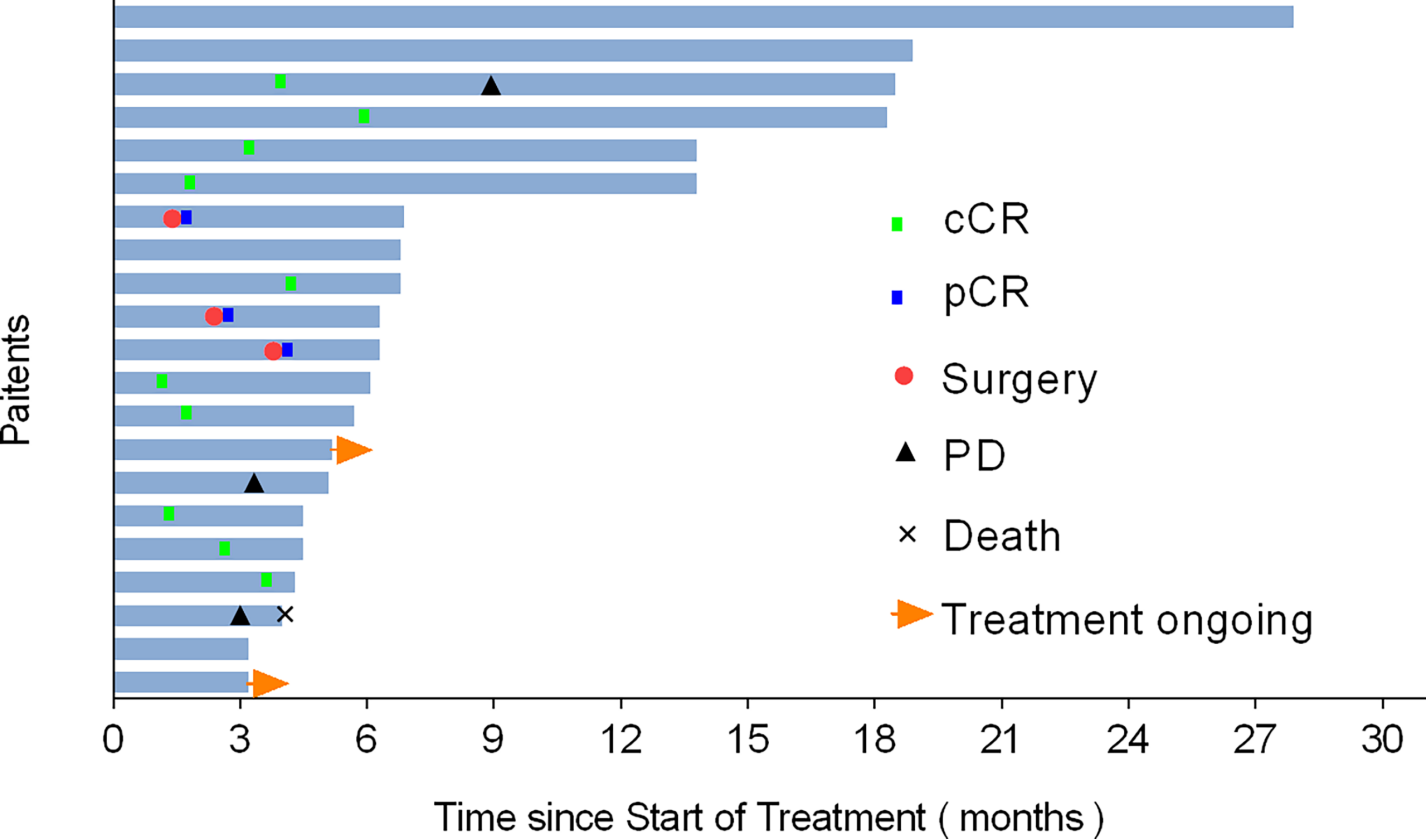
Swimmer plot representing critical points and duration of response (DOR) during treatment and follow-up, in patients who achieved complete response (CR) or partial response (PR), based on RECIST v1.1. Length of bars represents duration from initial H101 treatment to final follow-up.

Of the 7 patients treated with H101 monotherapy for local injection, 5 achieved LC and were free of relapse by the analysis deadline, with DOR ranging from 1.0 to 4.2 months; One patient received PR and was still under observation at the end of analysis with a shrinking trend; One patient developed PD at 6.9 months of follow-up. All these 7 patients were locally advanced cervical cancer patients with persistent lesions after concurrent chemoradiotherapy. Among all the patients enrolled, the longest PR time was followed up for 27.9 months and maintained PFS. Additionally, a CR patient remained disease-free even at a follow-up of 18.3 months, when data collection ceased. **Figure 3** showed typical cases. The patient with stage IB3 (FIGO 2018) at the initial diagnosis of mucinous adenocarcinoma of the cervix had a huge residual mass of cervix after non-standard treatment and two patients with locally advanced (IIIB FIGO 2018) cervical carcinoma had failed LC after concurrent chemoradiotherapy. Then, they experienced sufficient tumor shrinkage during H101 treatment to allow for curative surgery. Pathological assessments confirmed negative margins among all of them, and the three patients remained recurrence-free after 2.6, 4.0, and 5.6 months of post-surgery follow-up, respectively (**Figure 4**).

**Figure 5:**
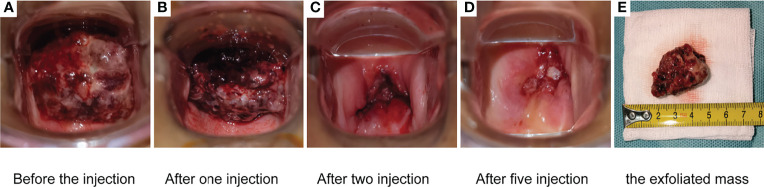
One patient with mucinous ovarian adenocarcinoma who had received five different chemotherapy regimens and 2 times of surgeries had huge metastatic carcinoma of vaginal cuff **(A)**. After one injection, the mass shrunk and showed necrosis **(B)**. The mass was partially exfoliated **(E)** and significantly reduced **(C)** after 2 injections. After completion of a course of injections (5.0 × 10^11^ virus particles daily for 5 days), vaginal cuff was only minimal residual **(D)**.

**Figure 6:**
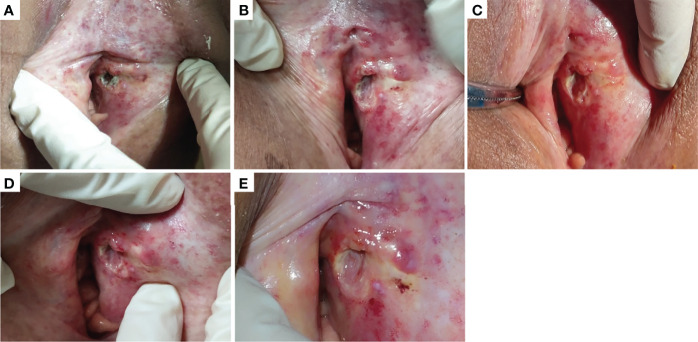
One patient with recurrent vulvar cancer had a lesion at left labia majora presented as an ulcer with foul pus on the surface **(A)**. With 5 days of continuous injection, the local state of the lesion gradually improved, and the ulcerated surface of the lesion was shallower and cleaner. **(B–D)** showed the changes of lesions after the first, third and fifth injection in the injection cycle, respectively. **(E)** showed the lesion in outpatient review after 1 week of this cycle of treatment.

**Figure 7:**
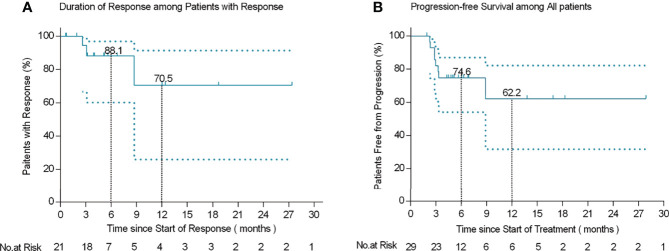
Kaplan-Meier curves of response duration among 21 patients who responded to treatment **(A)** and progression-free survival in 29 patients **(B)**.

Among the 21 patients who responded to the treatment, significant tumor regression and reduction in necrotic tissue were observed within 5 days of initial treatment. Typically, one patient with mucinous ovarian adenocarcinoma who had received five different chemotherapy regimens and 2 times of surgeries had huge metastatic carcinoma of vaginal cuff (**Figure 5A**). After one injection of H101, the tumor mass shrunk and showed necrosis (**Figure 5B**), and then the mass was partially exfoliated (**Figure 5E**) and significantly reduced (**Figure 5C**) after 2 injections. Notably, after completion of a course of injections (5 times), vaginal cuff was only minimal residual (**Figure 5D**). Besides, one patient with recurrent vulvar cancer had a lesion at left labia majora presented as an ulcer with foul pus on the surface. After 5 days of continuous injection, the local state of the lesion gradually improved, and the ulcerated surface of the lesion was shallower and cleaner (**Figure 6**). The median DOR had not been reached after median follow-up response duration of 3.9 months (range, 0.6 + to 27.4 + [plus signs indicate ongoing response at the time of analysis data cut-off]). The 6- and 12-month response rates were 88.1% and 70.5%, respectively (**Figure 7A**). These patients also did not reach median PFS after a median follow-up of 6.3 months (range, 3.2 + to 27.9 + [plus signs indicate continuing follow-up at the time of data analysis cut-off]). Their 6- and 12-month PFS rates were 74.6% and 62.2%, respectively (**Figure 7B**). We could not estimate OS because only one death occurred during follow-up by the time of data cut-off.

### Safety

The AEs are listed in **Table 3**. The most common AEs were fever (70%), followed by fatigue (37.9%), pain at the injection site (27.6%), hematology (27.6%), gastrointestinal reactions (nausea/vomiting, 24.1%), influenza-like symptoms (24.1%), intestinal fistula (3.4%) and bleeding at the injection site (3.4%). Of all these events (63), the majorities (90.5%) were grade 1 or 2, and the AEs with clinically significant were uncommon (7.9% grade 3 and 1.6% grade 4). Grade 3 AEs included rectovaginal fistula (1 case), severe fever (2 cases), influenza-like symptoms (1 case) and myelosuppression (1 case). The latter case involved a patient with mucinous ovarian adenocarcinoma who had received five different chemotherapy regimens; during our treatment, the individual developed grade IV myelosuppression. No treatment-related deaths occurred.

**Table 3:** Treatment-related Adverse Event.

Event	Grades No. (%)			
	G1-2	G3	G4	Any Grade
Fever	18 (62.1)	2 (6.8)	0 (0)	20 (70.0)
Flu-like symptoms	6 (20.7)	1 (3.4)	0 (0)	7 (24.1)
Nausea/vomiting	7 (24.1)	0 (0)	0 (0)	7 (24.1)
Fatigue	11 (37.9)	0 (0)	0 (0)	11 (37.9)
Injection site bleeding	1 (3.4)	0 (0)	0 (0)	1 (3.4)
Injection site pain	8 (27.6)	0 (0)	0 (0)	8 (27.6)
Intestinal fistula	0 (0)	1 (3.4)	0 (0)	1 (3.4)
Myelosuppression	6 (20.1)	1 (3.4)	1 (3.4)	8 (27.6)

## Discussion

Persistent, recurrent, and metastatic gynecological tumors are mostly treated with platinum-containing systemic therapy (3, 5, 28). However, the effective rate of treatment is limited, and the prognosis is poor (1–4). For localized and isolated lesions, LC has a significant impact on disease control and survival. Here, our study introduced a model with topical H101 as the main regimen and found that it showed anti-tumor activity with generally manageable safety in our test population. To the best of our knowledge, this study was the first to explore oncolytic viruses in gynecologic malignancies.

Of our 29 patients, 13 patients achieved LC, reaching a 44.8% rate in three months. Furthermore, 21 patients responded to treatment, achieving 72.4% ORR. These positive outcomes were consistent with previous studies in other cancers (29). Indeed, our treatment led to an even higher CR. It is worth mentioning that the single-agent application of H101 caused LC in 5 of the 29 patients in the study, and the single-agent LC rate was 17.2% without recurrence by the analysis deadline, demonstrating the effectiveness of H101 itself in this population. The LC rate increased to 44.8% after the combination of other treatments, demonstrating the necessity of combination therapy. Among the 13 patients who achieved LC, 5 of them with small residual lesions (<2 cm) received 1-2 courses of H101 treatment alone, and 5 with medium (2-4 cm) residual lesions were treated with H101 combined brachytherapy or external-beam radiotherapy (EBRT). The remaining 3 patients had large residual tumors (>4 cm) after radical chemoradiotherapy and received H101 intratumor injections. This intervention caused further tumor regression and created conditions for surgery, eventually leading to pathological CR. Our findings indicated that residual tumor size may be an important reference for choosing combination therapy. Up to the end of our follow-up period, none of the LC patients relapsed, except for 1 patient. She developed PD from pulmonary metastasis at 8.9 months follow-up. However, her local cervical lesion remained free of recurrence until the study ended (18.5 months follow-up).

All except 4 of the patients had received radiotherapy before recurrence, and 6 of them had received a second-course radiotherapy, which means that they may not be sensitive to radiotherapy. However, the ORR in our study was 72.4%, suggesting that H101 may increase the radiotherapy sensitivity. A prior study revealed that combining high doses of H101 and radiation yielded a synergistic anticancer effect in all tested cervical cancer cell lines (30). These outcomes are in line with research showing that OVs block DNA damage repair, sensitizing infected tumor cells to radiation and enhancing viral replication (22). Another *in vivo* test demonstrated that constant radiation combined with virotherapy eliminates cancer better than decay radiation and external beam radiation (31). In comparison, our study subjected 5 of 13 LC patients to combination H101 + brachytherapy at a relatively high radiation dose (four cases received interstitial brachytherapy). Therefore, H101 combined with radiotherapy may be a promising alternative for treating refractory gynecological malignancies. 4 patients who could no longer receive radiotherapy based on previous treatment history, local injection of H101 monotherapy may be an option. Additionally, patients with large local lesions may consider H101 injection to cause tumor regression before receiving brachytherapy (including interstitial needles); the combination of these two procedures should protect organs at risk of overdose while providing adequate radiation to the tumor, as demonstrated previously (32).

Notably, our target lesions were all accessible with the naked eye or palpation, enabling precise intratumor injection and high maneuverability. In the 21 responsive patients, we observed that tumor regression and reduction of necrotic tissue often occurred within the first five days of initial treatment. This encouraging progress led to great confidence for clinicians and patients, spurring the latter are compliance and promoting further treatment. The easily accessible tumors may thus be another possible reason for the beneficial effects observed in these patients.

Fever (70%) was the most common treatment-related side effect, occurring in a dose-depending manner consistent with other studies (33). In most patients, fever developed 1-24 h after H101 injection. Fever at 39°C was a self-limiting condition, while fevers higher than 39°C were treated with ibuprofen. 3 patients had grade 3 fever or flu-like symptoms that were effectively controlled with oral ibuprofen before injection and they generally recovered within 24 h. Other AEs included nausea/vomiting, fatigue, injection-site bleeding, and pain, but they all were transient and mild. However, 1 patient notably exhibited grade 4 thrombocytopenia after 10 days of H101 treatment. This individual had ovarian cancer and received 15 courses of chemotherapy plus two surgeries. The thrombocytopenia of grade ≥ 3 had also been reported in previous reports on treating ovarian cancer (34). Therefore, it cannot be determined whether the chemotherapy or H101 caused the grade 4 toxicity. In addition, 1 patient developed a rectovaginal fistula after H101 combined brachytherapy. This severe side-effect was first reported after OVs therapy, and radical chemoradiotherapy did not abolish the patient’s sizeable residual tumor. Although gynecological examination revealed “frozen pelvic change,” two more courses of H101 rapidly reduced the tumor. This significant tumor regression may be the main cause of fistula. Thus, chemoradiotherapy, targeted therapy, and H101 treatment together may be associated with adverse outcomes (35, 36).

## Conclusion

Our study provided evidence that H101 monotherapy or combination therapy is safe and effective for treating patients with recurrent, refractory, metastatic gynecological malignancies. However, this study has some limitations. First, this study was a retrospective study, and there may be selection bias. Second, the sample size of this study was small. In the future, we aim to expand our sample size and collect tissue samples for fine-tuned analysis. The clinical trial has already been registered (ClinicalTrials.gov identifier: NCT05051696), aiming to evaluate the efficacy and safety of H101 intratumor injection combined with or without radiotherapy in refractory or recurrent gynecological malignancies, and further research the mechanism of H101.

## Data Availability Statement

The original contributions presented in the study are included in the article/supplementary material. Further inquiries can be directed to the corresponding author.

## Ethics Statement

The study was approved by the Ethics Committee of The First Affiliated Hospital of Xi’an Jiaotong University, and informed consent was obtained from each participant. The patients/participants provided their written informed consent to participate in this study.

## Author Contributions

JZ and QZ conceived the study. JZ and QZ performed the literature search and writing of the manuscript. ZL, JW, and FS analyzed and interpreted the data. JS, TW, and FW collected and assembled the data. ZL submitted the manuscript and is the corresponding author. All authors contributed to the article and approved the submitted version.

## Conflict of Interest

The authors declare that the research was conducted in the absence of any commercial or financial relationships that could be construed as a potential conflict of interest.

## Publisher’s Note

All claims expressed in this article are solely those of the authors and do not necessarily represent those of their affiliated organizations, or those of the publisher, the editors and the reviewers. Any product that may be evaluated in this article, or claim that may be made by its manufacturer, is not guaranteed or endorsed by the publisher.
